# Attitudes toward Female Genital Mutilation/Circumcision: A Systematic Review and Meta-Analysis

**DOI:** 10.3390/healthcare9091184

**Published:** 2021-09-08

**Authors:** Leila Jahangiry, Tahereh Pashaei, Koen Ponnet

**Affiliations:** 1 Research Center for Evidence Based Medicine, Tabriz University of Medical Sciences, Tabriz 5166/15731, Iran; 2 Health Education and Health Promotion Department, Faculty of Health, Tabriz University of Medical Sciences, Tabriz 5166/15731, Iran; 3 Environmental Health Research Center, Research Institute for Health Development, Kurdistan University of Medical Sciences, Sanandaj 6617713446, Iran; pashaeit@gmail.com; 4 Faculty of Political and Social Sciences, IMEC-MICT, Ghent University, 9000 Ghent, Belgium; 5 Higher Institute for Family Sciences, Odisee, 1000 Brussels, Belgium

**Keywords:** female genital mutilation/circumcision (FGM/C), attitudes, girls

## Abstract

Background: Understanding the attitudes toward FGM/C held by people who have been involved in this practice can lead to more active interventions to prevent this harmful practice. In order to achieve this, a systematic review was performed on scientific articles. Methods: Electronic databases (PubMed, Scopus, and Science Direct) were examined to identify articles. Results: Our initial search resulted in 3013 articles, of which 40 articles with estimations of attitudes toward FGM/C were reviewed. The results indicate that the random-effects pooled estimation of negative attitudes toward FGM/C practice was 53% (95% CI 47–59; *p* < 0.001). Furthermore, the pooled estimation of attitudes toward the decision not to circumcise young daughters was 63% (95% CI 46–80; *p* < 0.001). Conclusion: Despite the increased awareness and efforts to ban FGM/C in many countries around the world, our review demonstrates that positive attitudes toward FGM/C are still far from being eradicated and have hardly changed in the past years. This issue reflects deeply rooted cultural and social concerns of health care professionals with regard to continuing the practice. The authors believe that circumcised women can play a key role in encouraging the abandonment of FGM/C through educational and cultural campaigns.

## 1. Background

Female genital mutilation/circumcision (FGM/C), or female circumcision, refers to all intentional acts that partially or totally remove the external female genitalia or female genital organs of young girls for cultural, traditional, or nonmedical reasons [1,2]. It is estimated that currently more than 200 million girls and women have undergone FGM in countries where this practice is endemic [3]. Recent studies indicate that FGM/C still occurs throughout Africa, the Middle East, and Asia [4]. FGM/C can have serious adverse effects on the physical and mental health of women in both the short and long term [5]. In the short term, excessive bleeding, shock, genital tissue swelling, fever, infection, and problems with urination and wound healing are the most common issues associated with female genital mutilation. The long-term physical effects of FGM/C include genitourinary infections (chronic pelvic infections, reproductive tract infections, genital infections, and vaginitis) and painful sexual intercourse [6]. One way of eliminating FGM/C is providing appropriate knowledge about FGM/C to the people who are involved in this practice, taking into account their sociocultural and personal sensitivities [7], although FGM/C has already endured for centuries because of tradition and culture [8]. Equipping people with information about the disadvantages of FGM/C remains crucial to alter their attitudes [9]. Furthermore, the literature provides evidence that the practice of FGM/C is performed in every social stratum, among both rich and poor people, educated and uneducated, as well as in both urban and rural regions. There is, however, evidence that women in the middle economic range are more likely to report themselves as having had FGM/C [10].

FGM/C is mostly carried out among countries in Africa, Asia, and Middle East. Studies show that the prevalence of FGM/C varies by region and ethnicity [11]. Regional location and ethnicity has an important role in women circumcision status. For example, a study conducted in northern Ghana, Bawku municipality reported a high prevalence of FGM/C (82%), while overall prevalence of FGM/C in Ghana is 4% [12]. 

As FGM/C is a cultural practice, efforts to end it require understanding the beliefs, attitudes, and perceptions that have sustained this practice over the centuries [13,14]. In particular, better understanding of whether or not attitudes toward FGM/C have changed over the years could help organizations develop strategies to encourage abandonment of FGM/C, and it could also help provide health planners with fundamental knowledge for developing strategies that might reduce FGM/C. Therefore, the purpose of this study is to perform a systematic review of the attitudes toward FGM/C among people who are involved in the practice.

## 2. Materials and Methods

### 2.1. Search Strategy

A systematic review of attitudes toward FGM/C was conducted using the Preferred Reporting Items for Systematic Reviews and Meta-analysis (PRISMA) guidelines [15]. Cross-sectional studies investigating the attitudes of FGM/C were examined. The search was done by two experienced researchers. The international electronic databases (PubMed, Scopus, and Science Direct) were searched for English-language peer-reviewed journal articles published between 1978 (i.e., the first published article retrieved on the topic) and 22 August 2021. The combination of following terms were used: (female$, or wom#n, or girl), AND (mutilation$, or circumcis$, or removal$, or alteration$, or cutting$, or clitorectom$, or infibulate$), AND (attitude$, or belief$, or opinion$, or perception$, or intention$). The first author of this article (LJ) manually screened the bibliographies of the retrieved articles for terms related to FGM/C and included these studies for the systematic review.

### 2.2. Inclusion and Exclusion Criteria

**Figure 1:**
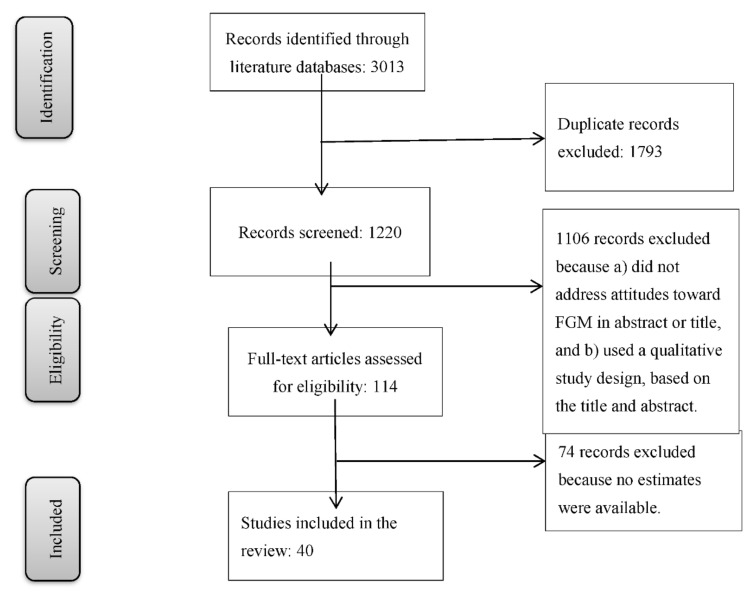
Number of articles eligible for the study.

Two researchers (LJ and TP) analyzed the search outcomes to find potentially eligible studies. A total of 3013 studies were retrieved from the three scientific databases for analysis. After screening the titles and abstracts for duplicates, 1793 articles were excluded. The remaining 1220 full-text articles were analyzed using the following criteria. First, only full-text articles reporting on quantitative studies were included. Abstracts, conference proceedings, commentaries, editorials, and qualitative articles were not eligible for further review. Second, articles had to relate to our research question and discuss the links between attitudes and FGM/C. Studies that only reported on the prevalence of FGM/C in certain populations were not included, as they did not address the research question. This resulted in the exclusion of 1106 records. The remaining 114 studies were considered for full-text review, of which 74 were excluded because they reported no estimations of attitudes toward FGM/C. The following statements were considered: (a) negative attitudes toward FGM/C, defined as FGM/C being harmful, having a negative impact on health, or causing complications, and (b) intentions to genitally mutilate daughters. Forty studies [10,16,17,18,19,20,21,22,23,24,25,26,27,28,29,30,31,32,33,34,35,36,37,38,39,40,41,42,43,44,45,46,47,48,49,50,51,52,53,54] were selected for the systematic review. Studies with two or more independent strata were considered separate studies [22,26,35,47]. Finally, 48 datasets from the 40 studies were extracted for meta-analysis. See Figure 1 for an overview.

### 2.3. Data Extraction and Quality Assessments 

A data-extraction sheet was developed in Microsoft Excel. Characteristics of the studies that were included are as follows: first author of the article, publication year, participants’ country of origin (Africa, Asia, Europe, USA, or Australia), type of participants (health care professionals, women with FGM/C, students, general population), participants’ gender and mean age, sample size, study design (cross-sectional or longitudinal), and the proportion (%) of negative attitudes toward FGM/C.

The quality of the studies was assessed by the Newcastle–Ottawa Scale (NOS). The NOS is a nine-item scale that scores articles based on three aspects: (a) sampling (representativeness of the sample, sample size estimation, nonresponse, and ascertainment of the exposure); (b) comparability (control for main factor and control for any additional factors); and (c) outcome (independent blind assessment, record linkage, and statistical test). Total NOS scores can range between 0 (lowest score) and 9 (highest) [55].

### 2.4. Statistical Analysis

For each dataset, the percentage (%) of respondents’ negative attitudes toward FGM/C was examined. Existence of heterogeneity was tested using Cochran’s Q-test at *p* < 0.05 level of significance. The *I*^2^ test was also used to calculate the percentage of heterogeneity [56]. A fixed-effects model was used to estimate pooled effect sizes. To investigate the source of heterogeneity, predefined subgroup analyses were performed using the type of respondents (i.e., students, health care professionals like midwives and nurses, general population, or women with FGM/C), participants’ country, and the NOS quality score. Publication bias was analyzed by funnel plot analysis and Egger’s regression asymmetry test [57]. All of the analyses were performed using STATA version 12.0 (Stata Corporation, College Station, TX, USA), and *p*-values below 0.05 were considered significant.

## 3. Results

### 3.1. Study Characteristics 

The characteristics of the included articles are presented in Table 1. The articles were published between 1978 and 2021 (22 August), with only two studies before 2000 (one article was published in 1978, and one in 1997). With regard to the FGM/C participants of the 40 studies, ten studies were conducted among health care professionals [21,23,31,34,35,36,41,45,50,51], eight were conducted among women from the general population [20,22,24,27,29,47,49,52], nine were conducted among students [10,17,30,32,33,38,40,43,46], four were conducted among a general population in which no distinction was made between men and women [26,28,48,53], four were conducted among circumcised women [18,19,39,42,54], one was conducted among online users [44], one was conducted among pregnant women [25], one among patients in hospitals [37], and one study was conducted among school teachers [16]. The sample sizes of participants varied from 63 to 21,756, with a total sample size of 184,574 participants. The age of the participants ranged between 15 and 60 years. The participants from the included studies were from 16 different countries or regions, including Egypt [10,18,19,24,29,32,43,47,53], Nigeria [16,17,20,25,40,41,50], Ethiopia [27,46,52], Sudan [21,28,30,38,48], Iraq [49], Australia [45], Kenya [37,42,54], USA [31], Yemen [22], Belgium [23,36], Gambia [33,34], Guinea [26], various African countries, [26] Middle East countries, [44], Iran [51], and Tanzania [39]. All studies used a cross-sectional design, and 11 of them were obtained from national Demographic and Health Survey (DHS) studies, like the Department of International Development Sudan Opinion Poll (DFIDSOP) dataset [28], Yemen Demographic and Health Survey (YDHS) [22], Egypt Demographic and Health Survey (EDHS) [19,24,47], Kenya Demographic and Health Survey (KDHS) [42], and the Global Online Sexuality Survey (GOSS) [44].

Quality scores ranged from 1 to 6. Seven studies had a quality score of 6, 11 studies had a quality score of 5, 18 studies had a quality score of 4, 3 studies had quality scores of 3 or 2, and 3 studies had a quality score of 1.

### 3.2. Meta-Analyses of Attitudes toward FGM/C

In this review, the dependent variable in the study is the percentage of participants who have negative attitudes toward FGM/C. The attitudes were calculated based on the 48 datasets from the cross-sectional and cohort studies that were conducted on health care professionals, students, and women with and without FGM/C. Two statements were considered in the assessments of attitudes toward FGM/C: (a) negative attitudes toward the practice of FGM/C in general; and (b) negative attitudes toward the decision to circumcise daughters now or in the future (18 of the 48 studies). Examples of statements of having negative attitudes toward the practice of FGM/C are as follows: women with FGM/C are at risk for gynecological complications [40], FGM/C causes anxiety disorders [37,38], FGM/C causes infection [42], FGM/C is not a good practice [43,52,53], FGM/C should be stopped [17,34], FGM/C is an illegal practice [10,25,26,27,50,51], FGM/C should be discontinued [22,26,28,38,47,48,54], one should oppose FGM/C [18,19,43], FGM/C can lead to girls’ deaths [18,32], and it is important to abandon FGM/C [39].

**Figure 2:**
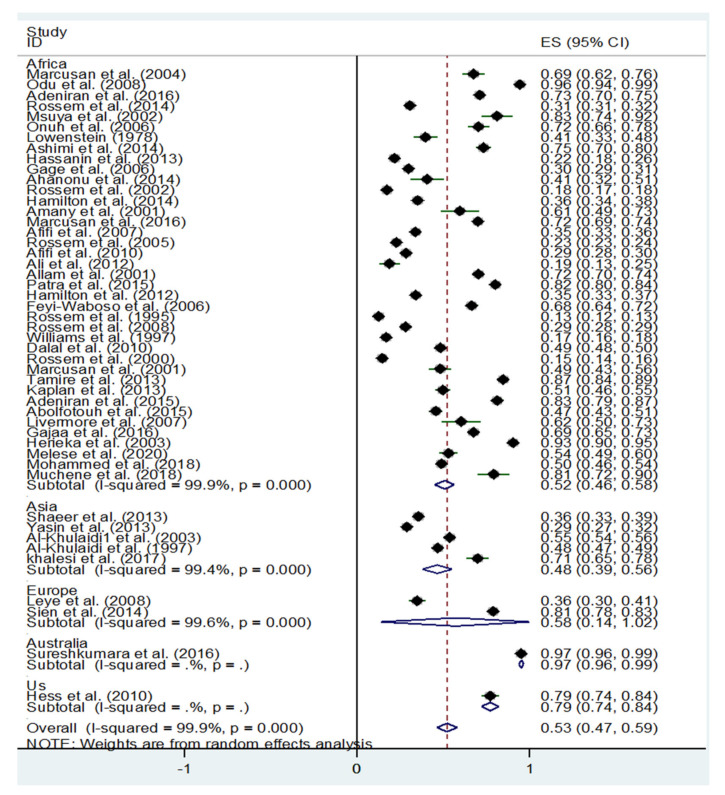
Negative attitudes toward FGM.

**Figure 3:**
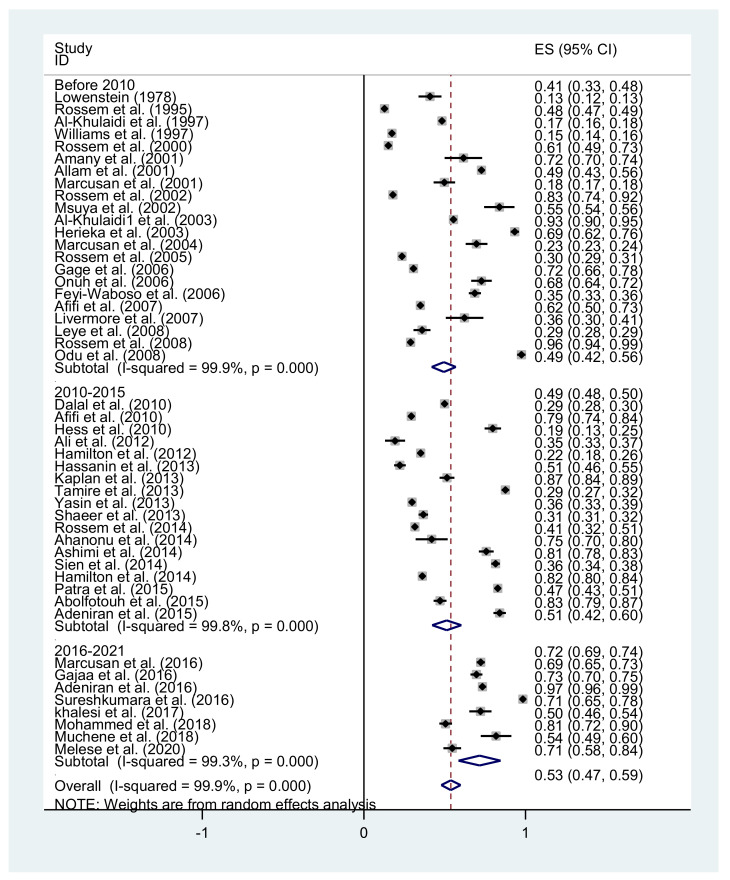
Negative attitudes toward FGM based on years categorizing.

Figure 2 provides a forest plot of the 48 studies, including the percentage of participants from each sample who had negative attitudes toward FGM/C as well as the 95% confidence intervals (CIs). Given that the cultural expectations and exposure towards FGM/C are different depending on the region where people live, we present the results separately for African countries, US, European, and Asian countries. The overall random-effects pooled estimation of persons with a negative attitude toward FGM/C was 53% (95% CI 47–59; *p* < 0.001), with a significant and high level of heterogeneity (*I*^2^ = 99.9%; *p* < 0.001). Because of the large amount of time between the first study and the last, we categorized the studies by those published before 2010, 2010 to 2015, and after 2016 to 2021. The random-effects pooled estimation of persons with a negative attitude toward FGM/C before 2010 was 49% (95% CI 42–56; *p* < 0.001), 2010 to 2015 was 51% (95% CI 42–60; *p* < 0.001), and after 2015 to 2021, 22 August, this estimation was 71% (95% CI 58–84; *p* < 0.001; *I*^2^ = 99.9%; *p* < 0.001), indicating that people have more negative attitudes toward FGM/C after 2015 than before (Figure 3).

**Figure 4:**
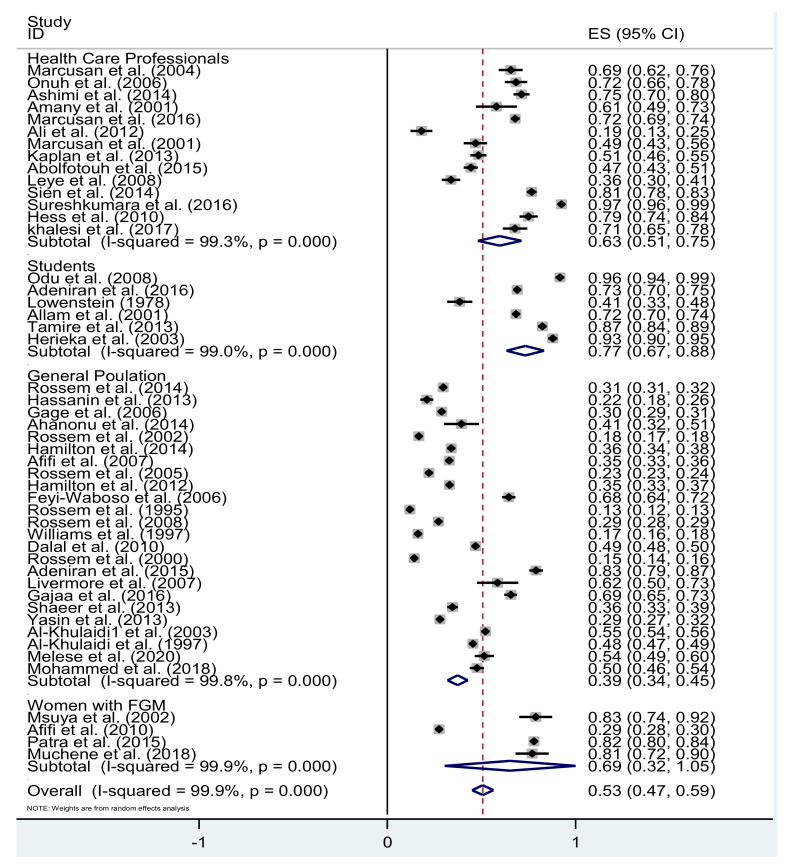
Negative attitudes toward FGM according to type of respondent.

**Figure 5:**
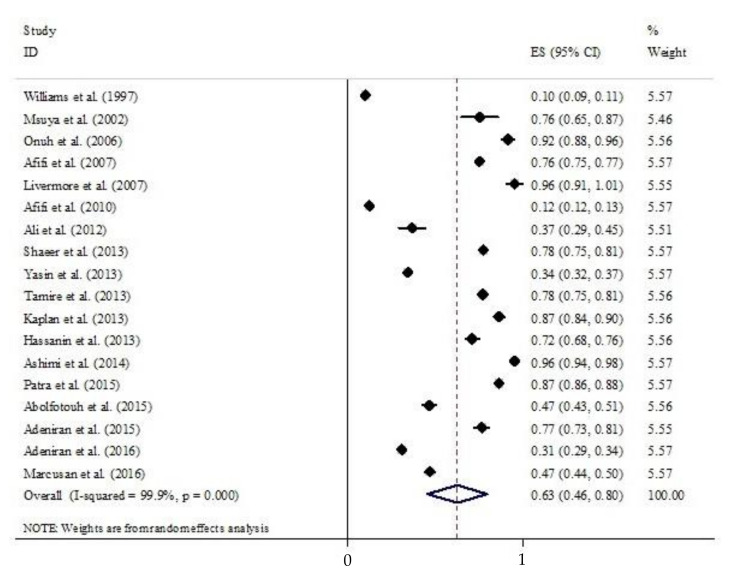
Attitudes toward circumcising daughters now or in the future.

A forest plot of the studies included in the meta-analysis arranged by type of participants and publication year is presented in Figure 4 (*I*^2^ 99.9%, *p* = 0.001). Figure 5 provides a forest plot of the 18 studies that reported the proportion (%) of attitudes toward the decision not to circumcise daughters now or in the future, together with the 95% CIs. Overall, 63% (95% CI 46–80; *p* < 0.001) of the participants confirmed that they would not circumcise their daughters now or in the future. There was significant heterogeneity between the studies (test for heterogeneity: *p* < 0.001 and *I*^2^ = 99.8%).

### 3.3. Sensitivity Analysis

Table 2 shows the results based on the attitudes toward FGM/C according to subgroup analyses to explore the origin of the heterogeneity between the studies. The overall random-effects pooled estimates were 0.63 for health care professionals (95% CI 0.49–0.75, *p* < 0.001), 0.77 for students (95% CI 0.66–0.87; *p* < 0.001), 0.69 for women with FGM/C (95% CI 0.32–1.05; *p* < 0.001), and 0.39 for the general population (95% CI 0.34–0.45; *p* < 0.001), indicating that students had the greatest proportion of negative attitudes toward FGM/C. Also, women with FGM/C had higher proportion of negative attitudes than health care professionals. The overall random-effects pooled estimates for the groups with respect to region were for 0.52 African countries (95% CI 0.46–0.58; *p* < 0.001), 0.48 for Asian countries (95% CI 0.39–0.56; *p* < 0.001), 0.58 for European countries (95% CI 0.14–1.1; *p* < 0.001), 0.79 for USA (95% CI 0.73–0.84; *p* < 0.001), and 0.97 for Australia (95% CI 0.96–0.98; *p* < 0.001).

Table 3 shows the results of the 18 studies that reported the proportion (%) of attitudes toward the decision not to circumcise their daughters. The overall random-effects pooled estimate for health care professionals was the highest, at 0.69 (95% CI 0.51–0.87; *p* < 0.001), and the estimate for the students was the lowest, at 0.54 (95% CI 0.08–1.0; *p* < 0.001). This indicates that health care professionals had the highest proportion of negative attitudes toward circumcising their daughters now or in the future. The overall random-effects pooled estimates for subgroups based on countries were 0.51 for Asian countries (95% CI 0.50–0.53; *p* < 0.001) and 0.33 for African countries (95% CI 0.32–0.34; *p* < 0.001). Also, the studies with lower quality scores had higher pooled estimates, at 0.66 (95% CI 0.38–0.93; *p* < 0.001), compared to those with higher quality scores, at 0.59 (95% CI 0.32–0.86; *p* < 0.001).

### 3.4. Publication Bias

**Figure 6:**
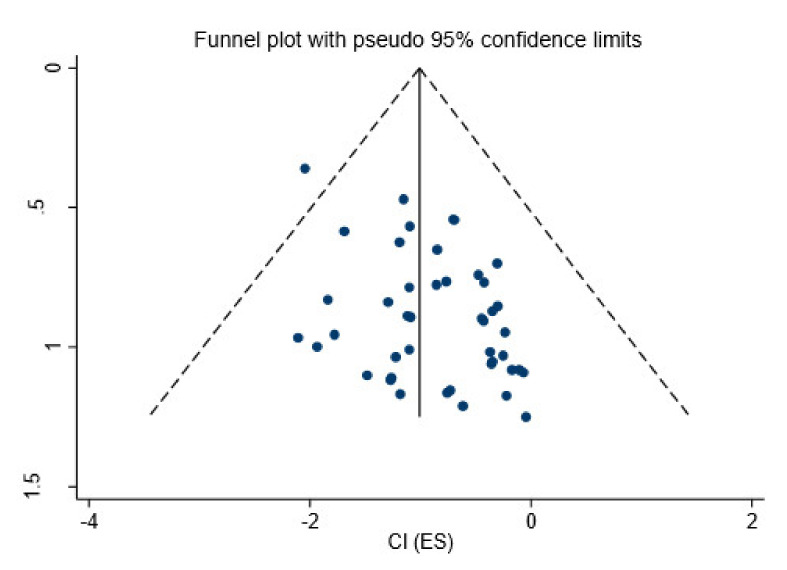
Funnel plot.

Publication bias was highlighted and graphically confirmed by the funnel plots. The funnel plots in Figure 6 show no publication bias among the studies, with the highest-precision studies plotted near the average and distributed symmetrically about the mean. Large studies are shown at the top of the graph, and smaller studies are shown at the bottom.

## 4. Discussion

This systematic review aimed to assess the attitudes toward FGM/C between the first study published on this topic in 1978 and studies published till August 22, 2021. The results of this study indicate that approximately 50% of the total participants across all of the studies reviewed believe that FGM/C is not a harmful practice for women. Looking at all studies published between 2010 to 2015, still around 51% of participants had negative attitudes toward FGM/C. Also, more than 60% of the general population and about 40% of health care professionals show negative attitudes toward FGM/C. The results demonstrate that despite many efforts to ban FGM/C in countries around the world, positive attitudes toward FGM/C are still far from being eradicated and have hardly changed over the past decades. Therefore, to eradicate the practice of FGM/C, a major attitudinal change is required.

It is interesting that from 1978 to 1995 there was only one study that investigated attitudes toward FGM/C (with inclusion of estimates). The rapid increase in studies on attitudes toward FGM/C after 2000 shows that FGM/C is an important problem that has gained increased attention worldwide. UNICEF’s 2016 report highlights that health care providers perform FGM/C due to erroneous information [58,59]. This is consistent with our finding that 37% of health care professionals are willing to perform FGM/C. One explanation for this is that FGM/C is mostly carried out by traditional circumcisers, who often play other central roles in communities, such as attending childbirth [60]. Our findings suggest that health care professionals do not consider the adverse consequences of FGM/C and insist on continuing this practice for sociocultural reasons rather than for reasons related to health care. This issue reflects deeply rooted cultural and social concerns among health care professionals with regard to continuing the practice.

Our results further revealed that women with FGM/C were more likely to disapprove of the continuation of FGM/C. One plausible explanation is that circumcised women have experienced the harmful effects of FGM/C [24], and they are therefore well aware of the negative health consequences of the practice, like difficulties in pregnancy or sexual dissatisfaction. Therefore, circumcised women can play a key role in encouraging the abandonment of FGM/C. Women with FGM/C can act as communication channels for both training and educational programs, because their audience will be confronted with their real experiences of FGM/C. Women with FGM/C might have an impact on the communities in which they live by serving as role models for decision-makers, influencing policies and working collaboratively with organizations advocating for FGM/C eradication. Empowering women might be a solution, so that they also can help to correct misconceptions, guiding families, and especially young couples, and informing them about the adverse consequences of FGM/C.

Our findings also demonstrate that the majority of students have negative attitudes toward this practice. This can be explained by the fact that students are in an educational environment, and their knowledge and attitudes are affected by their general education [32]. Still, eliminating FGM/C is difficult because of the time it requires to change traditional beliefs and attitudes. A substantial effort to improve knowledge among FGM/C-practicing cultural groups seems to be necessary [40]. Previous studies have recommended that education on the harmful effects of FGM/C could deter people from advocating for the practice and help change beliefs in traditional cultural contexts [32,61].

Analyzing the 18 studies from 1978 to 2021 on people’s attitudes toward circumcising their own daughters now or in the future showed that approximately 40% of the participants considered performing this procedure on their daughters. In such a situation, health care professionals might be in a good position to inform people about the negative effects of FGM/C. To protect the next generation from the harmful impacts of FGM/C, Desrumaux and Ballo have suggested that a change might be possible by employing a social change strategy based on health promotion and human rights [62]. This strategy would require a long-term approach within the education system and could lead to a change social dynamics if a majority of women refuses to have their daughters circumcised. According to the authors of that study, both political and social actors have to be involved to change attitudes toward FGM/C, and education has to be translated into action by establishing new institutional structures within the community [63,64]. Social actors can promote the full participation of young people—and especially young men, whose role is essential in the transformative process—to create an environment that is favorable to change [65].

## 5. Limitations

Despite the interesting findings of this study, the first limitation of this study is that we only have data of some countries (e.g., Guinea and US) from a particular year. For these countries, the overall estimation is difficult to interpret. A second limitation is that a number of surveys from different countries are unfortunately not published as scientific articles and thus not included in this study. A third limitation of this study could be the time difference between the different studies. It is expected that the attitudes should have been increased by time, particularly during the last decade. We tried to account for this issue with dividing the studies to those before and after 2010 and analyzing them separately. A final limitation is that we did not take into account several sociodemographic variables (e.g., population density, religion), because this information was not always described in the studies. These factors might however further unravel why people have positive or negative attitudes toward FGM/C.

## 6. Conclusions

Despite many efforts to ban FGM/C in countries around the world, positive attitudes toward FGM/C are still far from being eradicated and have hardly changed, indicating that a major attitudinal change is required to eliminate this practice. This issue reflects deeply rooted cultural and social concerns among health care professionals with regard to continuing the practice. It seems that circumcised women can play a key role in encouraging the abandonment of FGM/C through educational and cultural campaigns.

## Figures and Tables

**Table 1:** Descriptive statistics of the included studies (*n* = 48).

	First Author of the Article	Year	Country	Study Sample	*N*	Quality	Study Design
1	Abolfotouh [10]	2015	Egypt	Students	600	5	Cross-sectional
2	Adeniran [16]	2015	Nigeria	School teachers	371	4	Cross-sectional
3	Adeniran [17]	2016	Nigeria	Secondary students	1536	4	Cross-sectional
4	Afifi [18]	2010	Egypt	Women with FGM	15,572	6	EDHS
5	Afifi [19]	2007	Egypt	Women with FGM	5613	5	DHS
6	Ahanonu [20]	2014	Nigeria	Women	95	2	Cross-sectional
7	Ali [21]	2012	Sudan	Midwives	157	1	Cross-sectional
8	Allam [32]	2001	Egypt	University students	1700	4	Cross-sectional
9	Al-Khulaidi [22] *	1997	Yemen	Women	10,345	5	DHS
10	Al-Khulaidi [22] *	2013	Yemen	Women	11,252	5	DHS
11	Ashimi [50]	2014	Nigeria	Nurses	350	2	Cross-sectional
12	Cappon Sien [23]	2015	Belgium	Midwives	820	3	Cross-sectional
13	Dalal [24]	2010	Egypt	Women	9159	4	DHS
14	Feyi-Waboso [25]	2006	Nigeria	Pregnant women	600	4	Cross-sectional
15	Gage [26]	2006	Guinea	General population	8215	4	Cross-sectional
16	Gajaa [27]	2016	Ethiopia	Women	610	5	Cross-sectional
17	Hamilton [28] *	2012	Sudan	General population	2228	5	DFIDSOP
18	Hamilton [28] *	2014	Sudan	General population	2204	5	DFIDSOP
19	Hassanin [29]	2013	Egypt	Women	500	4	Cross-sectional
20	Herieka [30]	2003	Sudan	Students	414	4	Cross-sectional
21	Hess [31]	2010	US	Midwives	243	4	Cross-sectional
22	Kaplan [32]	2013	Gambia	Students	468	5	Cross-sectional
23	Khalesi [51]	2017	Iran	Midwives	168	4	Cross-sectional
24	Leye [36]	2008	Belgium	Gynecologists	333	4	Cross-sectional
25	Livermore [37]	2007	Kenya	Patients	68	4	Cross-sectional
26	Lowenstein [38]	1978	Sudan	Students	185	1	Cross-sectional
27	Marcusan [34] *	2001	Africa	Health professionals	225	5	Cross-sectional
28	Marcusan [34] *	2004	Africa	Health professionals	184	5	Cross-sectional
29	Marcusan [35]	2009	Gambia	Health professionals	1256	5	Cross-sectional
30	Melese [52]	2020	Ethiopia	women	325	6	Cross-sectional
31	Mohammed [53]	2018	Egypt	General population	618	6	Cross-sectional
32	Msuya [39]	2002	Tanzania	Women with FGM	63	4	Cross-sectional
33	Muchene [54]	2018	Kenya	Women with FGM	68	5	Cross-sectional
34	Odu [40]	2008	Nigeria	Female students	200	4	Cross-sectional
35	Onuh [41]	2006	Nigeria	Nurses	193	6	Cross-sectional
36	Patra [42]	2015	Kenya	Women with FGM	2284	4	KDHS
37	Refaat [43]	2001	Egypt	Students	69	1	Cross-sectional
38	Rossem [47] *	1995	Egypt	Women	14,769	6	EDSH
39	Rossem [47] *	2000	Egypt	Women	15,558	6	EDSH
40	Rossem [47] *	2003	Egypt	Women	9154	6	EDSH
41	Rossem [47] *	2005	Egypt	Women	19,461	6	EDSH
42	Rossem [47] *	2008	Egypt	Women	16,524	6	EDSH
43	Rossem [47] *	2014	Egypt	Women	21,756	6	EDSH
44	Shaeer [44]	2013	Middle East	Internet users	992	4	Online survey
45	Sureshkumara [45]	2016	Australia	Pediatricians	497	4	Cross-sectional
46	Tamire [46]	2013	Ethiopia	High school girls	780	6	Cross-sectional
47	Williams [48]	1997	Sudan	General population	3805	4	Cross-sectional
48	Yasin [49]	2013	Iraq	Women	1987	5	Cross-sectional

Notes: * The asterisks refer to studies with multiple datasets. EDHS: Egypt Demographic and Health Survey; DHS: Demographic and Health Survey; DFIDSOP: Department for International Development Sudan Opinion Poll; KDHS: Kenya Demographic and Health Survey.

**Table 2:** Findings of the subgroup analyses of negative attitudes toward FGM.

	No. of Studies	Pooled Estimates [95% CI]	I^2^	*p*-Value for Heterogeneity	Tau-Squared
**Participants**					
Health care professionals	15	0.63 [0.49–0.75]	99.3	<0.001	0.054
Women with FGM	3	0.69 [0.32–1.05]	99.9	<0.001	0.139
Students	6	0.77 [0.66–0.87]	98.9	<0.001	0.016
General population	23	0.39 [0.34–0.45]	99	<0.001	0.192
**Quality score**					
≤4	22	0.60 [0.48–0.73]	99.9	<0.001	0.091
>4	25	0.46 [0.40–0.52]	99.9	<0.001	0.022
**Country**					
African	38	0.52 [0.46–0.58]	99.9	<0.001	0.033
Asian	5	0.48 [0.39–0.56]	98.8	<0.001	0.009
European	2	0.58 [0.14–1.1]	98.8	<0.001	0.101

**Table 3:** Findings of the subgroup analyses of attitudes toward circumcising daughters now or in the future.

	No. of Studies	Pooled Estimates [95% CI]	I^2^	*p*-Value for Heterogeneity	Tau-Squared
**Participants**					
Health care professionals	7	0.69 [0.51–0.87]	99.5	<0.001	0.0581
Women with FGM	3	0.58 [−0.012–1.18]	100	<0.001	0.276
Students	2	0.54 [0.08–1.0]	99.8	<0.001	0.107
General population	6	0.61 [0.29–0.92]	99.9	<0.001	0.154
**Quality score**					
≤4	10	0.66 [0.38–0.93]	99.9	<0.001	0.192
>4	8	0.59 [0.32–0.86]	100	<0.001	0.153
**Regional subgroup**					
Africa	16	0.332 [0.32–0.34]	99.9	<0.001	<0.001
Asia	2	0.51 [0.50–0.53]	99.8	<0.001	<0.001

## Data Availability

The data collection tools and datasets generated and/or analyzed during the current study are available from the corresponding author on reasonable request.

## Abbreviations

FGM/C (Female Genital Mutilation/Circumcision); PRISMA (Preferred Reporting Items for Systematic Reviews and Meta-analysis); NOS (Newcastle–Ottawa Scale); DHS (Demographic and Health Survey); DFIDSOP (Department of International Development Sudan Opinion Poll); YDHS (Yemen Demographic and Health Survey); EDHS (Egypt Demographic and Health Survey); KDHS (Kenya Demographic and Health Survey); GOSS (Global Online Sexuality Survey); CIs (confidence intervals).
